# Dosage Forms Suitability in Pediatrics: Acceptability of Antibiotics in a German Hospital

**DOI:** 10.3390/antibiotics12121709

**Published:** 2023-12-07

**Authors:** Viviane Klingmann, Thibault Vallet, Juliane Münch, Lena Wolters, Robin Stegemann, Hans Martin Bosse, Fabrice Ruiz

**Affiliations:** 1 Department of General Pediatrics, Neonatology and Pediatric Cardiology, Medical Faculty, University Children’s Hospital Düsseldorf, Moorenstrasse 5, 40225 Düsseldorf, Germany; juliane.muench@med.uni-duesseldorf.de (J.M.); lena.wolters@hhu.de (L.W.); robin.stegemann@hhu.de (R.S.); hansmartin.bosse@med.uni-duesseldorf.de (H.M.B.); 2 ClinSearch, 110 Avenue Pierre Brossolette, 92240 Malakoff, France; thibault.vallet@clinsearch.net (T.V.); fabrice.ruiz@clinsearch.net (F.R.)

**Keywords:** pediatric drug formulation, drug administration, infants, toddlers, antibiotics, acceptability, swallowability, palatability

## Abstract

Although drug acceptability can have a significant impact on patient adherence in pediatric therapy, data are limited, even for common therapeutic areas. We present the second part of an acceptability study conducted at the University Children’s Hospital Düsseldorf, Germany. The study investigated the acceptability of most commonly used antibiotics in a pediatric hospital setting. The researchers used the acceptability reference framework to score the acceptability of five antibiotics based on 150 real-life observer reports of medicine intake. Four antibiotics assessed in this study were formulated as preparations for injection (ampicillin, ampicillin/sulbactam, ceftriaxone, and gentamicin) and one as a powder for oral liquid suspension (co-amoxiclav). All the antibiotics formulated as preparations for injection were rated negatively due to high rates of negative reactions (80%), the use of restraint (51%), the use of extra devices (99%), and long preparation and administration times (100%). The antibiotic formulated as a powder was significantly more well accepted. The study concluded that there is a lack of appropriate formulations for antibiotics for use in children. These findings are important in improving knowledge on acceptability drivers and might help in formulating and prescribing better medicines for children. The study highlights the need for healthcare professionals to have knowledge about the acceptability of different products to select the best-adapted product for each patient.

## 1. Introduction

The acceptability of dosage forms plays a decisive role in prescriptions, especially in pediatrics. Since children, depending on their age, may not yet be able to understand the rationale behind taking medication, it is important to make medications as suitable as possible for them. The active ingredient only reaches its site of action if the child accepts to take the medication. Acceptability, which is defined by the EMA (European Medicines Agency) as the “overall ability and willingness of the patient to use and its care giver to administer the medicine as intended”, is therefore a crucial criterion in drug administration in children [1].

The necessity of additional dosage forms for pediatrics, as well as the lack of pediatric studies, has already been pointed out in 2007 by the EMA’s Paediatric regulation [2]. In the 2013 EMA “Guideline on pharmaceutical development of medicines for pediatric use”, the agency demanded that “evaluation of patient acceptability of a paediatric preparation should be an integral part of pharmaceutical and clinical development” [1]. Following the EMA’s request, several new dosage forms for children were developed and evaluated in clinical trial and real-world evidence (RWE) study settings, by various researchers including the authors [3,4,5]. Another part of this study, focusing on the acceptability of analgesics and antipyretics, has already been published in the journal *Pharmaceutics* [6].

However, there is still little knowledge about the acceptability of the drugs already on the market. Even though 25% of all prescribed antibiotics are administered to children, there has not been adequate research on their acceptability in pediatrics so far, although it is the main factor for adherence in antibiotic treatment [1,7]. Non-adherence or the incorrect dosing of antibiotics can contribute to the development of antimicrobial resistance, and therefore, optimizing the use of antibiotics is one of the five strategic objectives in the WHO (World Health organization) Global action plan on antimicrobial resistance [8].

Acceptability is not only influenced by dosage forms, but also by several other characteristics of the product, the patient, and the setting of administration. Therefore, generic products may have different acceptability rates compared to the reference product. This could be due to altered tablet size, flavors, sweeteners, or coloring agents [1,9]. It is therefore important to know the acceptability of the drugs on the market, in order to use them appropriately. 

An observational multicentered international study performed by part of our research team has already revealed some acceptability drivers in commonly used oral antibiotics in children [5]. Although oral antibiotics are widely used, injectable formulations are also commonly used due to many advantages such as reliability in case of emergencies, avoiding interference by liver metabolism or overcoming individual issues such as uncooperative and vomiting patients. The acceptability of injectable drugs in pediatrics has become more significant recently. Although some aspects of acceptability, such as pain, have received substantial interest of researchers, there is a sparsity in the literature specifically addressing the assessment of injectable product acceptability [10].

In this study, acceptability of the most commonly prescribed antibiotics across all routes of administration in the Department of General Pediatrics, Neonatology and Pediatric Cardiology at the University Hospital Düsseldorf, Germany, was assessed in children aged 0–17 years. A proven data-driven approach, the CAST methodology [6,11], was used.

## 2. Materials and Methods

### 2.1. Study Design and Setting

This monocentric, cross-sectional, and observational study was approved by the Ethics Committee of the Medical Faculty of the Heinrich-Heine-University Düsseldorf, Germany (vote no. 2020-962, 23 July 2020). It was registered in the German Clinical Trial Register (No. DRKS00021800) and conducted according to the International Council for Harmonisation of Technical Requirements for Pharmaceuticals for Human Use’s standard of “Good Clinical Practice”. The research was conducted in the Department of General Pediatrics, Neonatology and Pediatric Cardiology of University Hospital Duesseldorf between August 2020 and June 2021.

### 2.2. Subjects

Eligible subjects were children aged 0–17 years who were treated with any of the following medications, with the exception of patients receiving intravenous medications if the intravenous device was already in place, as insertion of such a device was considered as part of the acceptability assessment. The antibiotics investigated are the antibiotics currently approved in Germany and most commonly used in inpatient and outpatient hospital settings. Therefore, most of the preparations were administered intravenously:

- Amoclav forte (co-amoxiclav) 250–62.5 mg/5 mL. Powder for oral suspension (Hexal^®^, Holzkirchen, Germany).
- Ceftriaxone 1 g (Hexal^®^, Holzkirchen, Germany) or 2 g (Hikma^®^, London, UK). Powder for suspension for injection.
- Ampicillin/sulbactam 1–0.5 g or 2–1 g. Powder for suspension for injection (Puren^®^).
- Ampicillin 0.5 g, 1 g or 2 g. Powder for suspension for injection (Ratiopharm^®^, Ulm, Germany).
- Refobacin (Gentamicin) 10 mg. Solution for injection (Merck^®^, Darmstadt, Germany).

Subjects were included on a voluntary basis, without randomization. A written consent by both parents was mandatory before starting any study-related procedures. A written assent from the patient was obtained where possible.

Thirty evaluations of each medicine were necessary for gaining reliable data on acceptability using the CAST methodology and consequently, 150 evaluable administrations were observed.

### 2.3. Data Collection

Once enrolled in the study, a standardized questionnaire was completed by a trained researcher observing the first administration of one of the medicines under investigation. 

The researcher reported the observations in Table 1.

Each evaluation corresponded to a specific combination of one observed measure for each of the nine observational variables. In addition to the observer-reported outcomes, information on the patient (e.g., age and sex), the prescribed treatments (e.g., the required dose, dosing frequency), and medicine use circumstances (e.g., time of administration) were collected.

### 2.4. Data Analysis

Acceptability scoring was performed using the CAST methodology. On May 2023, The EMA published a letter of support for CAST in relative acceptability testing for oral medicines in children under 12 years of age [12]. Herein we used the acceptability reference framework for research purposes only allowing to explore acceptability drivers. This model was based on a multivariate analysis of a large set of 3130 standardized real-life observer-reported outcomes, comprised of those collected in this sub-study and those similarly collected in various other countries since 2015. The published data analysis procedure—15 research articles between 2017 [11] and 2022 [6]—is succinctly described hereafter.

As a first step, multiple correspondence analysis was used to summarize the similarities between the ratings and the main relationships between these measures themselves in a three-dimensional map (3D map). Interpretation of the 3D map is based on distance between elements: proximities on the map express similarities. As the dimensions of the map revealed those associations and dissociations of observed measures that contributed the most to explaining variability observed in the large set of data, the map highlighted the major information in terms of medicine acceptability variation. Next, hierarchical clustering based on principal components and k-means consolidation grouped the ratings into two clusters according to Euclidean distances on the 3D map. The clusters described by the observed measures overrepresented in their subset defined two coherent and meaningful acceptance profiles. The “positively accepted” and the “negatively accepted” profiles were represented by green and red zones, respectively, on the 3D map.

All the evaluations collected in this study were plotted on the 3D map. The barycenter of the evaluations of a particular medicine defined its position on the 3D map. Confidence ellipses surrounding the barycenter for all dimension pairs (1–2, 1–3, and 2–3) defined an area containing its true position with 90% probability if the experiment were to be repeated. The final result of the algorithms was translated into a binary summary score as a first level of interpretation: if the barycenter, along with the entire confidence ellipsis surrounding it, fell within the green area of the 3D map, the medicine was classified as “positively accepted”. Using the CAST methodology, a minimum of 30 evaluations was required to obtain a reliable score with a satisfactory precision. Below this threshold, only acceptability tendency could be described.

Acceptability evaluation is relative and consequently, the comparison of antibiotic medicinal products within the reference framework is the second level of interpretation. Distinct acceptability scores were significantly different if their confidence ellipses did not overlap on the 3D-map.

To investigate what drives acceptability difference, the different components of the scores were studied as a third level of interpretation. The significance of the differences observed for the nine observational variables (e.g., result of intake, or patient’s reaction) was assessed using Pearson’s chi-squared test (χ^2^) or alternatively, Fisher’s exact test (F) when there was null expectation in the contingency table or few observations for individual fields of this table (less than expectation of 5 for 20%).

The significance of the differences observed in terms of patients’ characteristics (e.g., sex, age) was similarly assessed. 

Data analysis was performed using R version 1.0.136© (RStudio Team (2016). RStudio: Integrated Development for R. RStudio, Inc., Boston, MA, USA). The R packages FactoMineR [13] and missMDA [14] were used to perform multivariate analysis and to handle missing data, respectively.

## 3. Results

### 3.1. Study Subject

In this study, 150 evaluations were collected: 30 for each drug of interest. For 3 drugs formulated as powder for suspension for injection, preparations with different strengths were assessed: ceftriaxone 1 g (*n* = 18) and 2 g (*n* = 12); ampicillin/sulbactam 1–0.5 g (*n* = 22) and 2–1 g (*n* = 8); ampicillin 0.5 g (*n* = 24), 1 g (*n* = 4) and 2 g (*n* = 2).

Table 2 presents the characteristics of the 150 patients stratified by drugs. Forty-five percent were girls, and the mean age of the patients was 3.8 years (SD = 5.2, range 0–17). Most of the patients (62%) were less than 3 years of age, 13% were preschoolers, 9% grade-schoolers, and 16% were aged 12 years of age or older.

There was no significant difference between the patients treated with the oral preparation and those treated with injections in terms of sex (χ^2^: *p* = 0.44), age of patients (χ^2^: *p* = 0.27), and geographical origin of both parents (F: *p* = 0.67). However, while healthcare professionals oversaw the administration of all the preparations for injection, the oral liquid preparation was mainly administered by a caregiver, and occasionally by the patient themself (F: *p* < 0.001). Considering the preparations for injection, there were significant differences according to sex (χ^2^: *p* = 0.03) and age of patients (F: *p* = 0.029), between the patients treated with the different drugs. The patients treated with ampicillin and refobacin preparations tended to be younger than the average (<2 years) and most were boys. The dosages of ceftriaxone, amipicillin/sulbactam and ampicillin used increased correspondingly to the age of the children.

### 3.2. Acceptability

Among the 150 evaluations collected in this study, there were 32 distinct combinations of observed outcomes. Nine different combinations were reported for the assessments of all injectables. The following combination was used in almost half the cases (49%): “Fully taken”, “Negative reaction”, “Long time”, “No divided dose”, “No food drink”, “No alteration”, “Use extra device”, “No reward” and “Use restraint”. Twenty-five different combinations were reported for the 30 assessments of oral preparation of co-amoxiclav. This illustrates the variability of use under real-world conditions. The following combination reflecting medicine use without issue apart from preparation and administration time, was the most used (10%): “Fully taken”, “Neutral reaction”, “Long time”, “No divided dose”, “No food drink”, “No alteration”, “No extra device”, “No reward” and “No restraint”.

**Figure 1:**
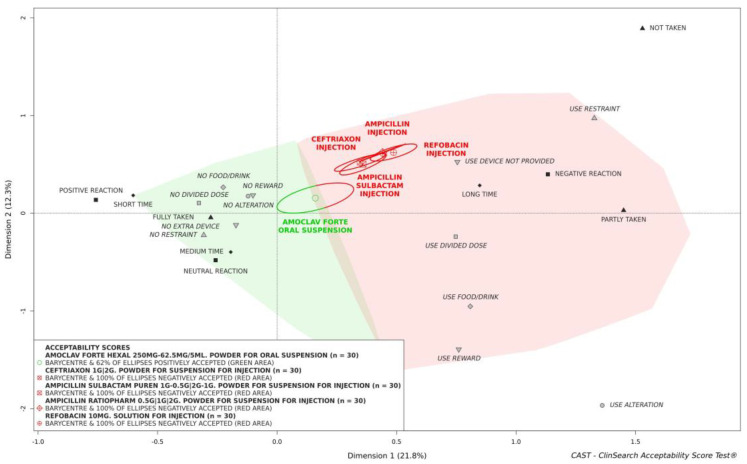
Acceptability scores of the five antibiotic medicinal products in pediatrics. Each antibiotic medicinal product was positioned onto the acceptability reference framework at the barycenter of its 30 evaluations. If the barycenter, along with the entire confidence ellipsis surrounding it, does not belong to the green area of the map, materializing “positively accepted” profile, the antibiotic medicinal product could not be classified as positively accepted.

Figure 1 presents the acceptability scores of the medicines of interest, and Table 3 itemizes the observer-reported outcomes composing these scores.

All preparations for injection were similarly negatively accepted as their barycenters, along with their entire confidence ellipses, are fully located in the red area of the 3D-map. There is no significant acceptability difference between these preparations as confidence ellipses overlap. This results from an absence of significant difference for all the nine observational variables. More negative reactions and use of restraints were reported for ampicillin and refobacin preparations used in younger patients in this study. However, these data are not significant. Consequently, the barycenters of these preparations are located farthest from the green area on the 3D map.

The barycenter of the evaluations of oral co-amoxiclav, along with 62% of the confidence ellipses surrounding it, is in the positively accepted area of the 3D map. However, this medicine cannot be classified as positively accepted due to a significant part of confidence ellipses being in the red area. Nonetheless, it was significantly better accepted than all the preparations for injection. Such acceptability variation between the oral preparation and the preparations for injection was due to significant differences for all the constituting observational variables apart from the result of the intake (F: *p* = 0.1) and using a reward (F: *p* = 0.58). There were significantly more negative reactions in patients treated with preparations for injection than in those treated with the oral preparation (χ^2^: *p* < 0.001). In addition, those patients receiving preparations for injection were more often forced to take their medication (χ^2^: *p* = 0.03). The preparation and administration time was longer for intravenous medication (F: *p* < 0.001) requiring the use of specific extra devices such as intravenous access, syringe, pump, pipe, needle, or minispike (χ^2^: *p* < 0.001). Dividing the intake of a required dose which cannot be taken as a whole (χ^2^: *p* < 0.001), using food or drink to mask the taste or ease swallowing (χ^2^: *p* < 0.001), or altering the medication use (F: *p* = 0.001) were the methods significantly more reported to ease or achieve administration of the oral preparation.

## 4. Discussion

In this study, we investigated the acceptability of the five antibiotics most often used at the University Children’s Hospital Duesseldorf. According to the acceptability reference framework, although the oral preparation under investigation was better accepted than the preparations for injections, none of the investigated medicines could be classified as positively accepted.

Although the barycenter of the evaluations of the oral preparations of co-amoxiclav is in the positively accepted area of the map, a significant part of confidence ellipses is in the red area of the 3D map. The acceptability of oral antibiotics in pediatrics is likely to be driven by many factors such as the age and sex of patients, previous exposure to treatment, place of administration, administration device, flavor agent in excipients and active pharmaceutical ingredient [5]. The age of children is a key factor, as there are more acceptability issues in younger children than in older ones [5,15]. In this study, most evaluations of the oral preparation of co-amoxiclav were collected in young children aged 0 to 2 years (57%). Only 10% were collected in adolescents from 12 years of age. This could be due to the fact that common infant infections, such as otitis media, are treated with this medication [16]. Furthermore, young children are known to be the highest users of antibiotics [17,18,19]. This may explain the large confidence ellipse, which reflects heterogeneity in the evaluations. Considering acceptability variation in subpopulations of patients depending on age, it appeared that acceptability of the preparation of co-amoxiclav tended to improve as children grew older (see Figure S1 and Table S1). However, additional evaluations are needed to obtain robust results. 

This study was conducted at a hospital. The setting might have positively impacted the acceptability of oral liquid preparations of antibiotics in children due to healthcare professional skills. This bias does not seem to apply to newborns and infants [5]. Co-amoxiclav can be considered as a mid-range medication in terms of palatability, with a better taste than antibiotics such as cefpodoxime or clarithromycin [5,20,21]. However, previous studies have demonstrated the higher palatability or acceptability of antibiotics such as azithromycin [22], cefdinir [23], or cefixime [24,25,26,27]. Co-amoxiclav was flavored with lemon, peach–apricot and orange. Other preparations available on the German market are formulated with different aromas such as “strawberry”, “caramel, strawberry”, “lemon, peach, strawberry”, and “golden syrup, orange, raspberry”. In previous studies, the strawberry flavor was accepted positively by boys and girls; lemon-flavored antibiotics had a contrasting effect on boys and girls as they were positively accepted in boys and negatively accepted in girls. Banana-flavored medication was negatively accepted by both sexes. Lemon–peach–apricot-flavored medication seemed to be less accepted than other flavors. However, we need to take into consideration that other flavors tended to be given more at home, so the setting might be a confounder [5]. It is also noteworthy that in our preliminary study on analgesics, significantly more restraints (30%) were required for the oral administration of orange-flavored paracetamol than for berry-flavored ibuprofen (10%) [6]. It is questionable whether the flavor additive or the taste of the active ingredient is the decisive factor here. As flavor is likely to be a key factor for oral medicine acceptability in pediatrics, the influence of flavoring agents’ variation on acceptability of co-amoxiclav preparations should be further investigated.

Most antibiotics were administered intravenously. This is likely to be due to the setting in a hospital. In doctor’s offices, oral antibiotics are prescribed more frequently. As we chose the most common administrations of antibiotics in our setting, we compared only one oral solution to four i.v. administrations. These administrations are generally common worldwide and it is widely accepted that i.v.-administration, which is an invasive and potentially painful procedure, may have little acceptability in infants and children. On the other hand, this study is the first to quantify the burden of oral or i.v. antibiotics from the perspective of children and healthcare professionals.

In this study, all the preparations for injection were considered as poorly accepted, as their barycenter and the surrounding ellipses were fully located in the negatively accepted area of the 3D map. A likely reason for this might be the placement of the intravenous access necessary before administration of the drug. Additional to the pain during this procedure, a high needle fear prevalence in children could contribute to the negative reactions [28]. However, in our opinion, the establishment of intravenous access must also be part of the assessment of acceptability of a dosage form, as it is sometimes established exclusively for antibiotic administration. The results suggest that oral dosage forms are better accepted. It should therefore be evaluated in the individual case whether intravenous administration of an antibiotic and the associated placement of a peripheral venous access is absolutely necessary. Preparations of ceftriaxone, ampicillin, and ampicillin/sulbactam with a higher dosage tended to be closer to the positive area; however, there is a too-little number of evaluations per dosage to draw any sound conclusion. One reason for this might be that older children were mainly treated with these higher dosages (see Figures S2–S4 and Tables S2–S4). As for oral preparations, there is a tendency toward acceptability improvement as children grow older. However, preparations for injection seem to remain poorly accepted even in older patients (see Figure S5 and Table S5). Different aspects such as the formulation properties, dose volume, administration rate and duration, as well as site of injection, needle size or administration device, should be considered during development to improve the acceptability of parenteral formulations [10,29,30]. Despite a suboptimal acceptability, parenteral formulations allow drug administration for patients with serious illness or who have alteration of swallowing function. Furthermore, this route of administration allows the drug to be absorbed more rapidly, avoid the first-pass metabolism and is suitable for postoperative situations or emergency. Children who are admitted to the study hospital are often severely ill and thus require intravenous antibiotics. Due to this, mainly intravenously administered antibiotics were investigated.

## 5. Conclusions

The results of this study highlight the lack of appropriate formulations concerning antibiotics in children. It emphasizes the poor acceptability of intravenous dosage forms and encourages preference for other routes of drug delivery where feasible. The study contributes to the knowledge on acceptability drivers and hereby tries to improve formulations and medication for children.

## Figures and Tables

**Table 1:** Observer-reported outcomes describing the many aspects of acceptability.

Observational Variables	Observed Measures (Categories)
**Result intake** The results of intake of the required dose	Fully, Partly, or Not taken
**Patient reaction** The patient’s reaction to the intake on a 3-point facial hedonic scale	Positive, Neutral, or Negative reaction
**Preparation and administration time** The time hospital staff needed to prepare (from opening the packaging to having the required dose of medication ready to use, including all handling and modifications) and administer the required dose of medication (from the required dose of medication ready to use to the end of the intake), pooled and recoded	Short (≤1′), Medium (from 1′ to 2′30″), or Long (>2′30″) time
**Divided dose** Dividing the intake of a required dose which cannot be taken as a whole (e.g., successive sips of an oral liquid preparation, several tablets or pieces of tablets swallowed successively)	No divided dose, or Use divided dose
**Food/drink** Using food/drink (e.g., mixing with the drug or taking before/after to mask the taste or ease swallowing)	No food/drink, or Use food/drink
**Alteration** Altering the use, such as modifying the dosage form (e.g., prescribed dose of tablet split into fractions or crushed into powder) or using another route/mode of administration (e.g., oral administration of an injectable solution)	No alteration, or Use alteration
**Extra device** Using a device not provided with the medication (e.g., disposable spoon or oral syringe provided with another medication)	No extra device, or Use extra device
**Reward** Promising a reward	No reward, or Use reward
**Restraint** The child was made to take it	No restraint, or Use restraint

**Table 2:** Characteristics of the 150 patients included in the study, stratified by medicinal product.

Characteristics	Amoclav Forte 250–62.5 mg/5 mL. Powder for Oral Suspension (*n* = 30)	Ceftriaxone 1 g|2 g. Powder for Suspension for Injection (*n* = 30)	Ampicillin/Sulbactam 1–0.5 g|2 g–1 g. Powder for Suspension for Injection (*n* = 30)	Ampicillin 0.5 g|1 g|2 g. Powder for Suspension for Injection (*n* = 30)	Refobacin 10 mg. Solution for Injection (*n* = 30)
**Sex**					
Female	16 (53) ^a^	18 (60)	16 (53)	9 (30)	9 (30)
Male	14 (47)	12 (40)	14 (47)	21 (70)	21 (70)
**Age group**					
0–2 years	17 (57)	13 (43)	15 (50)	24 (80)	24 (80)
3–5 years	7 (23)	4 (13)	4 (13)	3 (10)	2 (7)
6–11 years	3 (10)	5 (17)	2 (7)	1 (3)	2 (7)
12–17 years	3 (10)	8 (27)	9 (30)	2 (7)	2 (7)
**Parents Geographical regions of origin**					
Western Europe	9 (30)	13 (43)	11 (37)	11 (37)	13 (43)
Central and Eastern Europe	7 (23)	4 (13)	7 (23)	6 (20)	6 (20)
Mixed origin including Western	5 (17)	5 (17)	4 (13)	4 (13)	3 (10)
Europe	3 (10)	4 (13)	1 (3)	4 (13)	2 (7)
Western Europe (no information for other parent)	3 (10)	1 (3)	2 (7)	1 (3)	1 (3)
Other <5% ^b^	3 (10)	3 (10)	5 (17)	4 (13)	5 (17)
**Person in charge of the medicine administration**					
The patient	6 (20)	0 (0)	0 (0)	0 (0)	0 (0)
A caregiver ^c^	15 (50)	0 (0)	0 (0)	0 (0)	0 (0)
A healthcare professional	9 (30)	30 (100)	30 (100)	30 (100)	30 (100)

^a^ *n* (%): number and percentages; ^b^ Other <5%: Northern Africa, Subsaharan Africa, Eastern Asia, Indian Subcontinent, Central Asia; ^c^ caregiver: family member, other adult helper.

**Table 3:** Observer-reported outcomes collected in this study for the five antibiotic medicinal products.

Observer-Reported Outcomes	Amoclav Forte 250–62.5 mg/5 mL. Powder for Oral Suspension (*n* = 30)	Ceftriaxone 1 g|2 g. Powder for Suspension for Injection (*n* = 30)	Ampicillin/Sulbactam 1–0.5 g|2–1 g. Powder for Suspension for Injection (*n* = 30)	Ampicillin 0.5 g|1 g|2 g. Powder for Suspension for Injection (*n* = 30)	Refobacin 10 mg. Solution for Injection (*n* = 30)
**Result intake**					
Fully taken	28 (93) ^a^	30 (100)	30 (100)	30 (100)	29 (97)
Partly taken	1 (3)	0 (0)	0 (0)	0 (0)	1 (3)
Not taken	1 (3)	0 (0)	0 (0)	0 (0)	0 (0)
**Patient reaction**					
Positive	5 (17)	1 (3)	0 (0)	0 (0)	0 (0)
Neutral	13 (43)	7 (23)	7 (23)	5 (17)	4 (13)
Negative	12 (40)	22 (73)	23 (77)	25 (83)	26 (87)
**Preparation and administration time**					
Short	4 (13)	0 (0)	0 (0)	0 (0)	0 (0)
Medium	5 (17)	0 (0)	0 (0)	0 (0)	0 (0)
Long	21 (70)	30 (100)	30 (100)	30 (100)	30 (100)
**Divided dose**					
No divided dose	24 (80)	30 (100)	30 (100)	30 (100)	29 (97)
Use divided dose	6 (20)	0 (0)	0 (0)	0 (0)	1 (3)
**Food/drink**					
No food/drink	24 (80)	30 (100)	30 (100)	30 (100)	30 (100)
Use food/drink	6 (20)	0 (0)	0 (0)	0 (0)	0 (0)
**Alteration**					
No alteration	26 (87)	30 (100)	30 (100)	30 (100)	30 (100)
Use alteration	4 (13)	0 (0)	0 (0)	0 (0)	0 (0)
**Extra device**					
No extra device	16 (53)	0 (0)	1 (3)	0 (0)	0 (0)
Use extra device	14 (47)	30 (100)	29 (97)	30 (100)	30 (100)
**Reward**					
No reward	30 (100)	29 (97)	28 (93)	30 (100)	29 (97)
Use reward	0 (0)	1 (3)	2 (7)	0 (0)	1 (3)
**Restraint**					
No restraint	22 (73)	18 (60)	18 (60)	12 (40)	11 (37)
Use restraint	8 (27)	12 (40)	12 (40)	18 (60)	19 (63)

^a^ *n* (%): number and percentages.

## Data Availability

Data underlying the study cannot be made publicly available due to legal and ethical considerations. European Union (GDPR) and French (Law no. 78–17 of 6 January 1978) laws restrict the public sharing of personally identifiable data. Requests for data will be processed according to the French MR-003 Code of conduct by the data controller, ClinSearch, which allows for the use of data for the purpose of reproducing study results. Requests to access the data for this purpose may be sent to the data protection officer of ClinSearch: dataprivacy@clinsearch.net and researchers outside the European Union will need to sign a transfer agreement.

## Supplementary Materials

The following supporting information can be downloaded at: https://www.mdpi.com/article/10.3390/antibiotics12121709/s1, Figure S1: Acceptability of Amoclav‘ forte 250 mg–62.5 mg/5 mL. Powder for oral suspension’ depending on age of patients; Figure S2: Acceptability of ‘Ampicillin. Powder for suspension for injection’ depending on strength (0.5 g, 1 g or 2 g); Figure S3: Acceptability of ‘Ampicillin/sulbactam. Powder for suspension for injection’ depending on strength (1 g–0.5 g or 2 g–1 g); Figure S4: Acceptability of ‘Ceftriaxone. Powder for suspension for injection’ depending on strength (1 g or 2 g); Figure S5: Acceptability of injections regardless of products depending on age of patients; Table S1: Differences observed in terms of observed-reported outcomes and patients’ characteristics for Figure S1; Table S2: Differences observed in terms of observed-reported outcomes and patients’ characteristics for Figure S2; Table S3: Differences observed in terms of observed-reported outcomes and patients’ characteristics for Figure S3; Table S4: Differences observed in terms of observed-reported outcomes and patients’ characteristics for Figure S4; Table S5: Differences observed in terms of observed-reported outcomes and patients’ characteristics for Figure S5.
