# Supplementary figures and images for: Dosage Forms Suitability in Pediatrics: Acceptability of Antibiotics in a German Hospital
Source: Antibiotics (Basel). 2023 Dec 7;12(12):1709. doi: 10.3390/antibiotics12121709 (PMC10740640; doi:10.3390/antibiotics12121709)

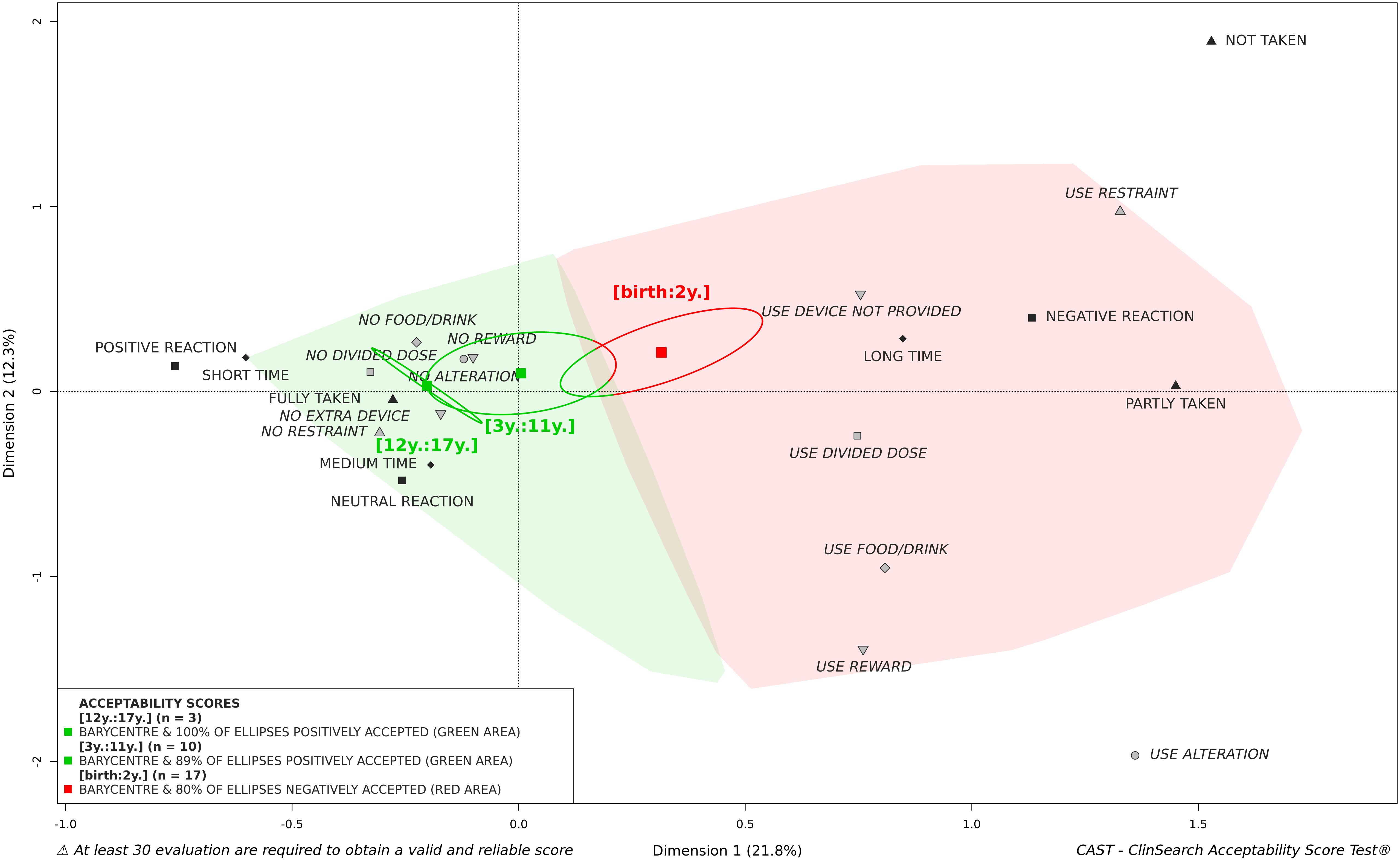

Supplement: Supplementary file 1 [file antibiotics-12-01709-s001.zip › Figure.S1.coamox.age.jpeg]

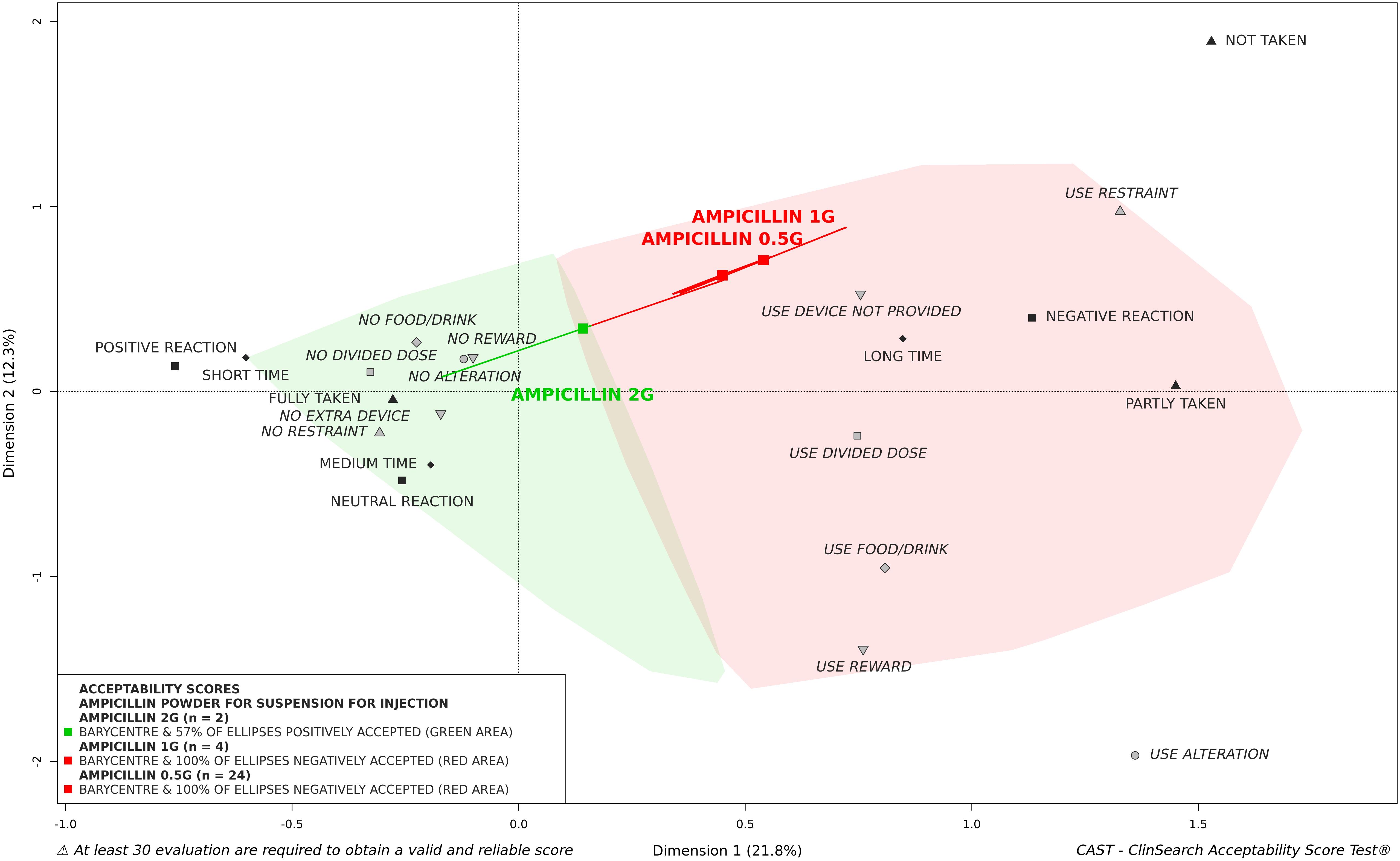

Supplement: Supplementary file 1 [file antibiotics-12-01709-s001.zip › Figure.S2.inj.ampicillin.jpeg]

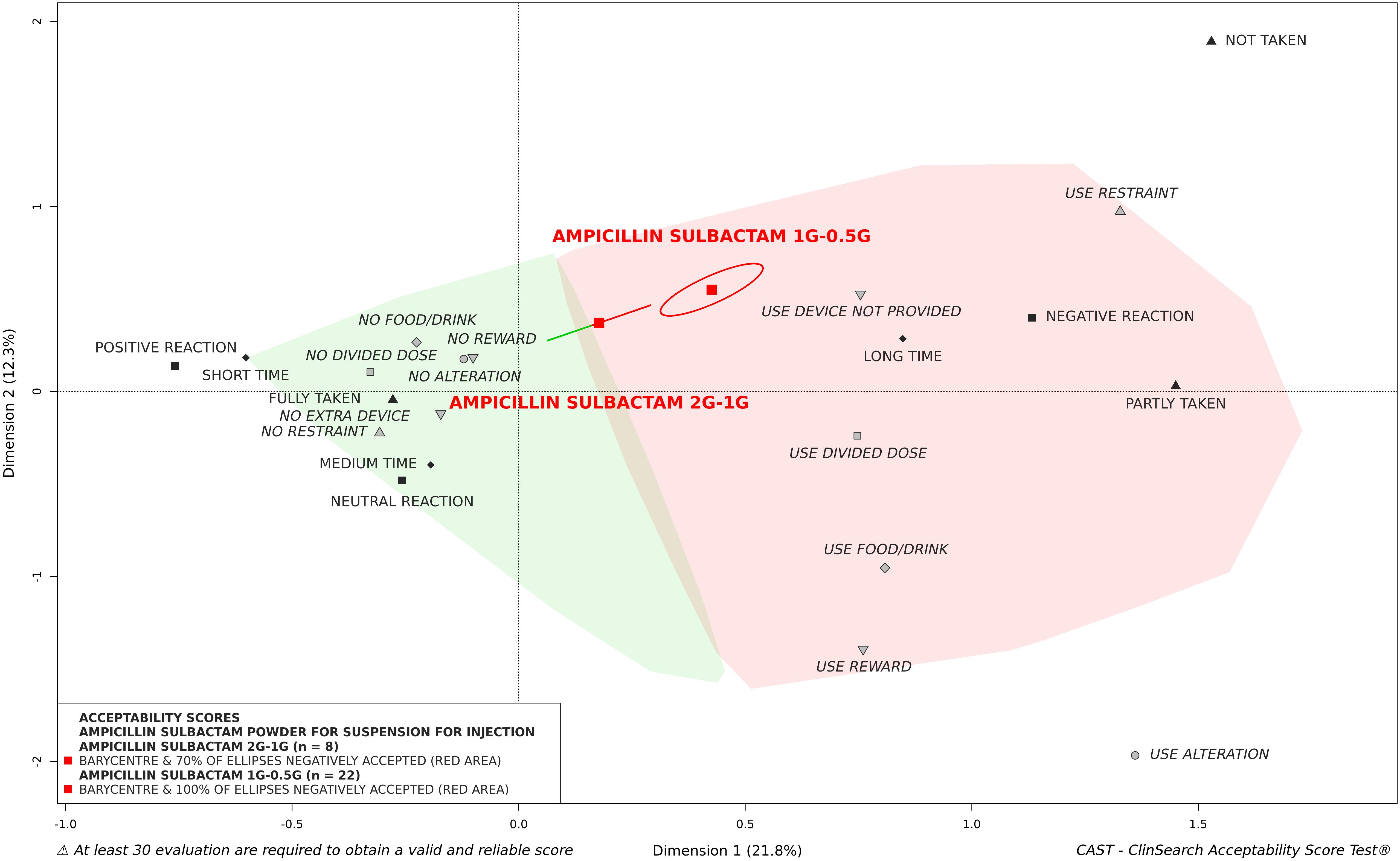

Supplement: Supplementary file 1 [file antibiotics-12-01709-s001.zip › Figure.S3.inj.ampicillinsulbactam.jpeg]

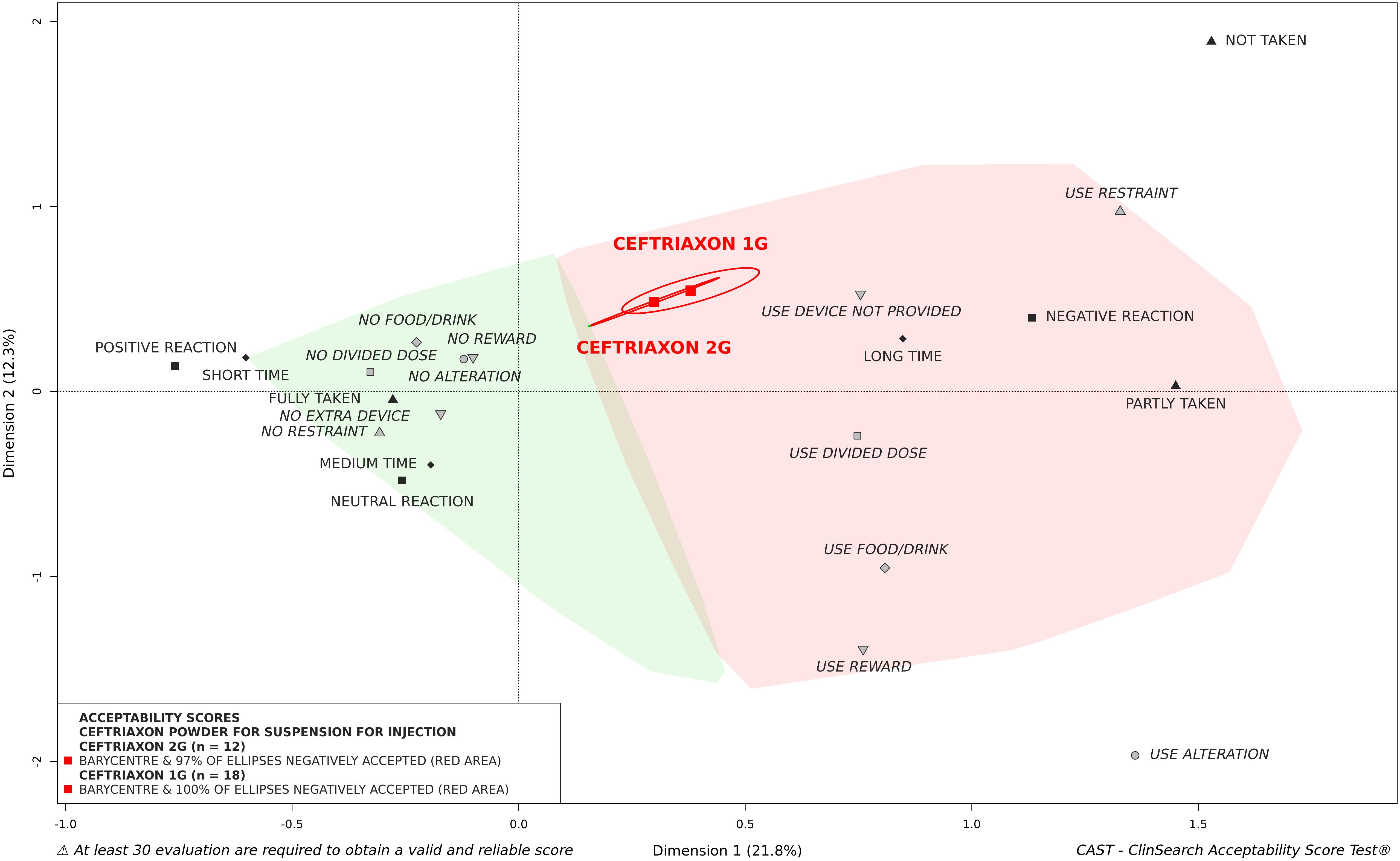

Supplement: Supplementary file 1 [file antibiotics-12-01709-s001.zip › Figure.S4.inj.ceftriaxon.jpeg]

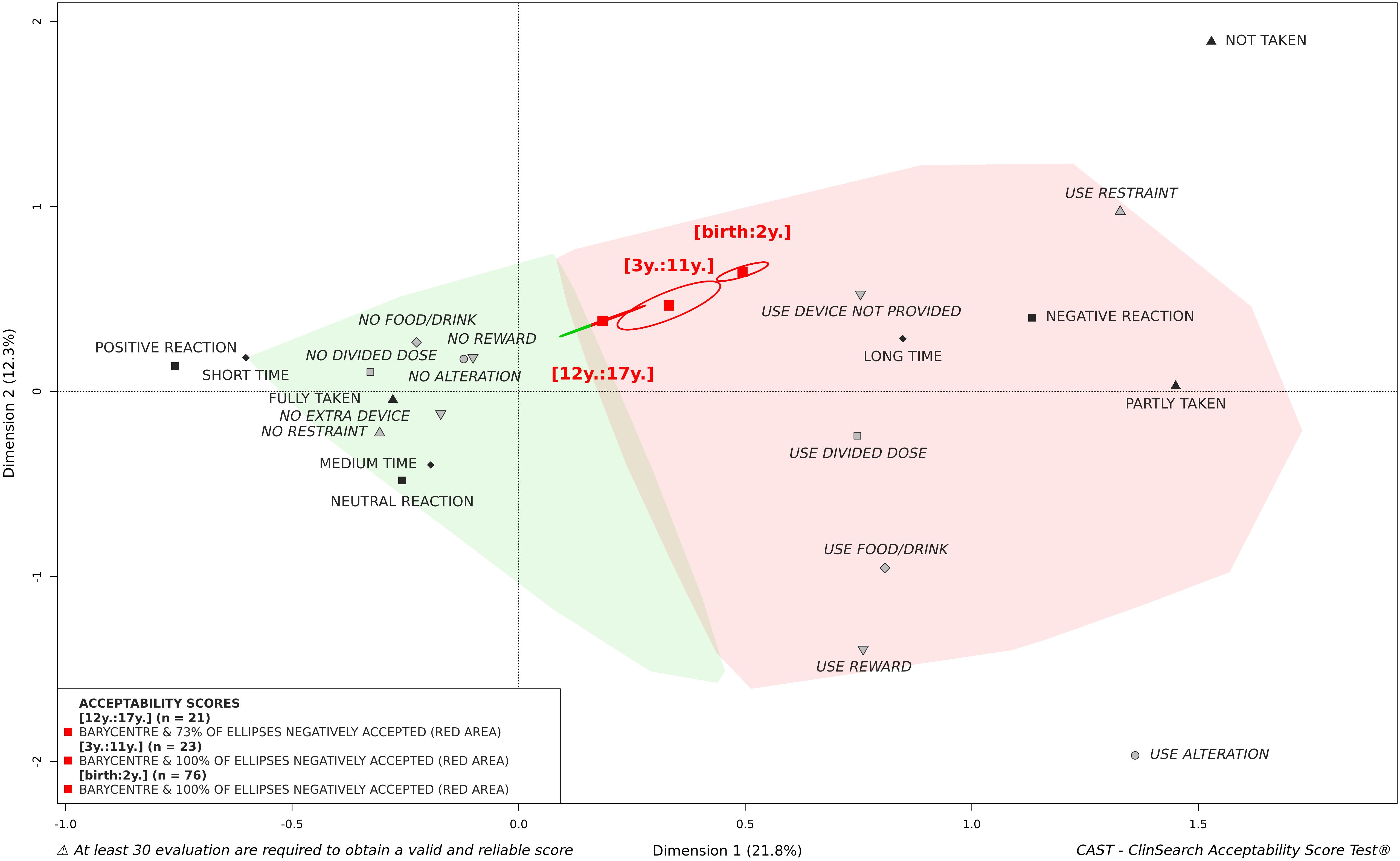

Supplement: Supplementary file 1 [file antibiotics-12-01709-s001.zip › Figure.S5.injection.age.jpeg]
